# Anti-melanogenesis effect from Wampee fruit pectin via α-MSH/TRY pathway in A375 cells

**DOI:** 10.1186/s12906-022-03646-6

**Published:** 2022-06-25

**Authors:** Weiyu Fu, Xuehua Liao, Qian Zhang, Yuzhen Zhu, Si Mei, Qian Li, Xin Zhou, Xiaojun Li, Hui Luo, Hua Ye, Kefeng Wu

**Affiliations:** 1 Marine Biomedical Research Institution, Guangdong Medical University, Zhanjiang, 524023 People’s Republic of China; 2 Guangdong (Zhanjiang) provincial laboratory of Southern Marine Science and Engineering, Guangzhou, 524023 People’s Republic of China; 3 Guangdong Key Laboratory for Research and Development of Natural Drugs, Guangdong Medical University, Zhanjiang, 524023 People’s Republic of China; 4 BoRui Saccharide Biotech Co. Ltd, Yangzhou, 225000 People’s Republic of China

**Keywords:** Wampee fruit pectin, Physicochemical characterization, Anti-melanogenesis, α-MSH/TRY pathway

## Abstract

**Background:**

Polysaccharides from wampee have been reported to process various biological activities, while the relationship between structure and bioactivities has been barely addressed. Pectin, an abundant water-soluble polysaccharide in wampee, showed significant antioxidant activity, which was associated with the anti-melanogenic activity. Therefore, this study investigated the physicochemical characteristics and the anti-melanogenesis effect of pectin extracted from wampee fruit in A375 cells.

**Methods:**

The physicochemical characterization of pectin from wampee fruit was investigated by gel chromatography (GCP), FT-IR spectroscopy, and NMR spectroscopy methods. The anti-melanogenesis effects and mechanism were evaluated by mushroom tyrosine enzyme and human melanin cell model in vitro.

**Results:**

The results showed that a molecular weight of 5.271 × 10^5^ Da wampee fruit pectin (WFP) were mainly composed of mannose (Man), ribose (Rib), rhamnose (Rha), glucuronic acid (Glc A), glucose (Glc), galacturonic acid (Gal A), galactose (Gal), and arabinose (Ara), which linked with →4)-β-D-Galp-(1 → units. The current study revealed that WFP could significantly suppress mushroom TRY activity in vitro. Furtherly, WFP significantly reduced intracellular and extracellular melanin formation in A375 melanoma cells depending on the presence of alpha-melanocyte stimulating hormone (α-MSH). TRY activity was only inhibited in α-MSH treated A375 cells. Western blot analysis demonstrated that WFP reverse α-MSH induced melanogenesis in A375 melanoma cells, including in down-regulated TRY, TYRP-1, TYRP-2, MITF and CREB expressions.

**Conclusion:**

These results indicated that WFP could inhibit α-MSH induced melanogenesis in A375 melanoma cells via α-MSH/TRY pathway. In conclusion, these data provided a new perspective to annotate WFP anti-melanogenesis activity mechanism.

## Introduction

Wampee (*Clausena lansium*) fruit, a special tropical fruit in the Southern region of China, contains abundant nutrition such as vitamins, minerals, organic acids, pectin, and other substances. Pectin is the major component that contributes more than 20% (w/w) of the total dry weight of fruits. It has been widely used in the cosmetics, food, and pharmaceutical industries due to its biocompatible and non-toxic properties. Recently, considerable attention has been focused on the bioactivity of pectin because it serves as a natural plant polysaccharide. Tremendous studies showed that pectin processed antioxidative [1], hypoglycemic [2], immunomodulatory [3], and anticancer activities [4], owing to the structural diversity of pectin. Pectin is a complex polysaccharide with a linear backbone of D-galacturonic acid (GA) units ploymerized by α-1,4-glucosidic bonds. Pectin has different branches containing large neutral sugars such as xylose, galactose, and arabinose [5, 6]. The diversity of pectin branches from various plant materials was reported to exhibit the different antioxidant and immunomodulatory activities [2]. Pectin as a primary water-soluble polysaccharide maybe plays an important role in antioxidant activity [7], while the anti-melanogenesis effect has been barely attention. . Therefore, analyzing the physicochemical characteristics and biological activity of the pectin is necessary for an in-depth discussion of the functionality and working mechanism of wampee fruit pectin (WFP).

Hyperpigmentation is a common skin disorder, which causes by the melanin overproduction in melanocytes. Melanogenesis comprises a complex regulated process by various factors including enzymes and hormones [8, 9]. Tyrosinase (TRY) is a key rate-limiting enzyme that could be activated by an increase of α-melanocyte stimulating hormone (α-MSH), and thus enhances the melanin synthesis processes [10, 11]. Specifically, α-MSH is originated from a precursor protein pro-opiomelanocortin (POMC), which could be produced by all skin cells including melanocytes, keratinocytes, and fibroblasts et al. The process of POMC cleavage was regulated by different factors such as ultraviolet irradiation, fluctuating hormones and immune response et al. [11].. Moreover, various literatures have revealed that α-MSH could upregulate TRY expression, which binds melanocortin 1 receptor (MC1R) to activate cyclic adenosine monophosphate (cAMP) [12]. Activation of cAMP increases microphthalmia-associated transcription factor (MITF) binding TRY promoter to stimulate melanogenesis via phosphorylation of cAMP-response element-binding protein (CREB) [13, 14]. Thus, the suppression of TRY and MITF is a critical strategy for developing novel hyperpigmentation agents.

Polysaccharides with antioxidant and anti-melanogenesis activity have been used as skin-whitening agents. However, lack of studies reports anti-melanogenesis activity caused by pectin polysaccharides, despite pectin having been used as a food additive and pharmaceutical excipients for some time. Therefore, the present study aimed to analyze the structure of WFP and investigate anti-melanogenesis activity and its mechanism by using α-MSH treated human melanoma cells A375, the results of which could be helpful in understanding structure-function relationships of wampee fruit pectin and the anti-melanogenesis mechanism via α-MSH/TRY pathway.

## Materials and methods

### Materials

Wampee (*Clausena lansium* (Lour.) Skeels) (identified by Dr. Zhanping Gou, Department of pharmacognosy, Guangdong Medical University, China) was purchased from the local wholesale market (Maoming, Guangdong, China). D-Galacturonic acid monohydrate standard (purity ≥97%) was purchased from Sigma company (St. Louis, USA) and dialysis bags were obtained from United States union carbonization (Connecticut, USA). Citric acid, hydrochloric acid, sulfuric acid, and anhydrous ethanol were obtained from Sinopharm group chemical reagent co. LTD (Shanghai, China). EDTA-2Na was purchased from a chemical reagent factory (Guangzhou, China). All reagents are analytical grade. Carbazole (purity ≥96%) was purchased from Aladdin biochemical technology co., LTD (Shanghai, China). α-MSH, tyrosinase from mushroom, and L-DOPA were purchased from Sigma company (St. Louis, USA). TRY, tyrosinase-related protein 1 (TYRP-1), tyrosinase-related protein 2 (TYRP-2) and MiTF primary antibodies were purchased from Abcam company (Cambridge, UK). Creb (Phosopho-S133) antibodies (pCREB) were purchased from Signalway Antibody company (Maryland, USA).

### Extraction and purification of WFP

The precipitation produced by anhydrous ethanol was recovered through centrifugation, and washed by different concentration of ethanol. WFP extracted was added with 75% ethanol to precipitate, centrifuged, and then dissolved in water and concentrated. Afterwards, the pectin solution was repeated freezing and thawing three times to remove protein and was used to verify protein removal by freeze-melt. Then the above solution was separated by dialysis bags for molecular weight gradient with a molecular weight cut off the above 300 kDa. It was extensively dialyzed at 4 °C distilled water for 72 h, and the dialysis water was changed three times a day [15, 16]. Subsequently, it was freeze-dried to get the purified pectin.

### Determination of molecular weight

Molecular weight (Mw) was determined by high-performance gel-permeation chromatography (HPGPC) on a Waters 515 HPLC system, which was equipped with a Wyatt Dawn Heleos11 laser light scattering instrument and an Optilab T-rex refractive index detector.

### Chemical composition analysis

Phenol-sulfuricacid colorimetric method described by Wu et al. [17] for determining the sugar content used D-glucose as a standard. Sulfuric acid carbazole colorimetric method described by Blumenkrantz et al. [18] for analyzing uronicacid content used galacturonic acid as a standard. The method of Bradford described by Sedmak et al. [19] for measuring protein content using bovine serum albumin as a standard.

### Estimating degree of esterification (DE)

The DE was determined by the titration method. The content of methoxyl in WFP was converted to the degree of esterification [20]. WFP (200 mg) was wetted with 2 mL ethanol and dissolved in 100 mL distilled water. After dissolving fully, 2 drops of reagent (phenolphthalein) were added, and the solution was titrated with NaOH (0.1 M). The result obtained at the end-point was recorded as the first titer (V_1_). After adding 20 mL NaOH (0.5 M) for 2.5 h, the solution was titrated with H_2_SO_4_ (1.0 M) until a pale pink color vanished. This used volume of NaOH was recorded as the second titer (V_2_).

The degree of esterification was calculated using Eq. (1).

1 $$\mathrm{Degree}\;\mathrm{of}\;\mathrm{pectin}\;\mathrm{esterification}\;\left(\%\right)=\left[100\left({\mathrm C}_1{\mathrm V}_1-{\mathrm C}_2{\mathrm V}_2\right)\times0.031\times100\right]/16.3{\mathrm W}_1$$

Where:

C_1_ = The concentration of standard base solution added during saponification.

V_1_ = The volume of standard alkali solution added during saponification.

C_2_ = The concentration of H_2_SO_4_ standard solution.

V_2_ = The volume of standard H_2_SO_4_ standard solution.

W_1_ = The pectin sample weight.

### Gel strength analysis

Gel strength was determined by the SAG method. The sag was measured according to the IFT method [21]. The gels of standard pectin were prepared according to the Beda MY method [22].

### FT-IR spectrometric analysis

Fourier-transform infrared (FT-IR) spectra of WFP were measured by the KBr method with an FT-IR spectrophotometer in the range of 500 ~ 4000 cm^− 1^. WFP (2 mg) was ground with dried KBr (140 mg) powder in a mortar and pressed into pellets for FT-IR measurement on an IRTracer-100 FT-IR spectrometer (SHIMADZU, Japan).

### Analysis of monosaccharide composition

The monosaccharide composition of the polysaccharide was determined by a pre-column derivatization process using high performance liquid chromatography (HPLC) with D-glucose as the standard [23]. In brief, samples (10 mg) were hydrolyzed with 4 M trifluoroacetic acid (TFA) at 120 °C. Further, excess TFA was removed with methanol. Afterward, the dry hydrolysate was added to NaOH (0.3 M) and methanolic PMP (0.5 M) and then was incubated at 70 °C for 1 h. After cooling to room temperature, it added HCl solution (0.3 M) to neutralize it. Then it added chloroform extraction for 3 times and merged the water layer followed by filtering (0.45 μm nylon filters) before determining neutral sugars by high-performance liquid chromatography (HPLC). The mobile phase (5 mM sulphuric acid) was set at a flow rate of 0.6 mL·min^− 1^ at 65 °C.

### NMR spectroscopy analysis

WFP (60 mg) was dissolved in 750 μL D_2_O, and then centrifuged at 9075 g for 5 min. The ^1^H NMR, ^13^C NMR, ^1^H-^1^H correlation spectroscopy (^1^H-^1^H COSY), and ^1^H/^13^C heteronuclear single quantum correlation (^1^H/^13^C-HSQC) were determined by an NMR spectrometer (DMX500, Bruker company, German).

### The inhibitory effect of pectin on TRY activity

WFP (20 μL/well) dissolved in distilled water, phosphate buffer saline (50 mmol/L, 100 μL/well, PBS), and L-DOPA (5 mmol/L, 60 μL/well) were incubated in 96-well plates for 10 min at 37 °C in an atmosphere of 5% CO_2_. Then the mushroom tyrosinase solution (100 U/mg, 40 μL/well) was added to mix rapidly and evenly, and incubated at 37 °C for 20 min. The absorbance was read at 475 nm on a microplate reader (Epoch, Bio-Tek company, USA).

The inhibition rate was calculated using Eq. (2).

2 $$\mathrm{The}\;\mathrm{inhibition}\;\mathrm{rate}\;\left(\%\right)=\left[1-\left({\mathrm A}_1-{\mathrm A}_2\right)/\left({\mathrm A}_3-{\mathrm A}_4\right)\right]\times100$$

### Analysis of the effect of melanin synthesis on A375 cells induced by α-MSH

#### Cell culture

A375 cells were purchased from the American Type Culture Collection (VA, USA), cultivated at 37 °C in an atmosphere of 5% CO_2,_ and maintained in Dulbecco’s Modified Eagle Medium/High glucose (DMEM/HG) with 10% fetal bovine serum (FBS) and antibiotics (0.2% gentamicin).

#### Cell viability assay

Cell viability was measured by using 3-(4,5-dimethylthiazolyl-2)-2,5- diphenyltetrazolium bromide (MTT, 5 mg/mL). In brief, A375 cells (5 × 10^3^/well) were incubated in 96-well plates in 200 mL of DMEM containing 10% FBS and placed overnight. After treatment with α-MSH (2 μg/mL) for 12 h, the cells were treated with 0.125, 0.25, 0.5, 1, 2 and 4 mg/mL of WFP respectively for 12 h. Subsequently, the cells were incubated with MTT solution (0.5 mg/mL) for 4 h at 37 °C. The medium was removed and DMSO (150 mL/well) was added. The absorbance was read at 490 nm on a microplate reader (Epoch, Bio-Tek company, USA).

#### Measurement of melanin content

Melanin content in A375 cells induced by α-MSH was measured by using NaOH assay. Briefly, A375 cells (5 × 10^5^/well) were incubated in 6-well plates overnight. The cells were stimulated with α-MSH (2 μg/mL) for 12 h and then maintained serial concentration for 12 h or 24 h. After the treatment, the cells were washed twice with PBS, detached with 0.25% Trypsin-EDTA solution, and subsequently centrifuged at 2700 g for 5 min. The cells were solubilized in 1 N NaOH containing 10% DMSO by boiling at 80 °C for 1.5 h. The absorbance was read at 470 nm on a microplate reader (Epoch, Bio-Tek company, USA).

#### Intracellular TRY activity

TRY activity in A375 cells was evaluated by measuring the oxidation rate of DL-dopa. A375 cells (5 × 10^4^/well) were incubated in 6-well plates for 12 h, The cells were stimulated with α-MSH (2 μg/mL) for 12 h, and then maintained in several medium containing a serial of concentration of wampee for 12 h. After treatment, the cells were washed twice with PBS and lysed with 1% Triton X-100/PBS. The cell lysates were centrifuged at 800 rpm for 5 min. After the cell lysates were quantified at protein levels and adjusted protein concentrations with 1% Triton X-100/PBS, the supernatants of each cell lysate (30 mg) were dissolved in 100 mL of sodium phosphate buffer (0.1 mM, pH 6.8) and mixed with 100 mL of L-DOPA (5 mM) in a 96-well plate. The mixture was incubated at 37 °C for 25 min in an atmosphere of avoided light and its absorbance was read at 450 nm on a microplate reader (Epoch, Bio-Tek company, USA).

#### Western blot analysis

After treating A375 cells with α-MSH (2 μg/mL) and several medium containing a serial of concentration of wampee for 24 h, the cells were washed twice with PBS and lysed with lysis buffer containing protease and phosphatase inhibitors. Protein concentrations were measured by using BCA assay. The samples with aliquots of (40 mg/sample) were resolved by 10% sodium dodecyl sulfate-polyacrylamide gel electrophoresis and transferred to polyvinylidene fluoride membranes. The membrane was blocked with 5% nonfat milk (w/v) for 2 h and incubated for about 14 h with primary antibodies. After treatment, the immunoblots were incubated with suitable secondary antibodies for 2 h and detected using an ECL luminescence reagent. The results were visualized and analyzed using Image J analysis software.

### Data and statistical analysis

The data was expressed as the mean ± standard deviation (SD) from three independent experiments. One-way analysis of variance (ANOVA) with Dunnett’s post-hoc test was performed with the statistical significance using the SPSS program version. *P < 0.05* level was considered as the significance of differences.

## Results

### Purification of WFP and physicochemical properties

**Fig. 1:**
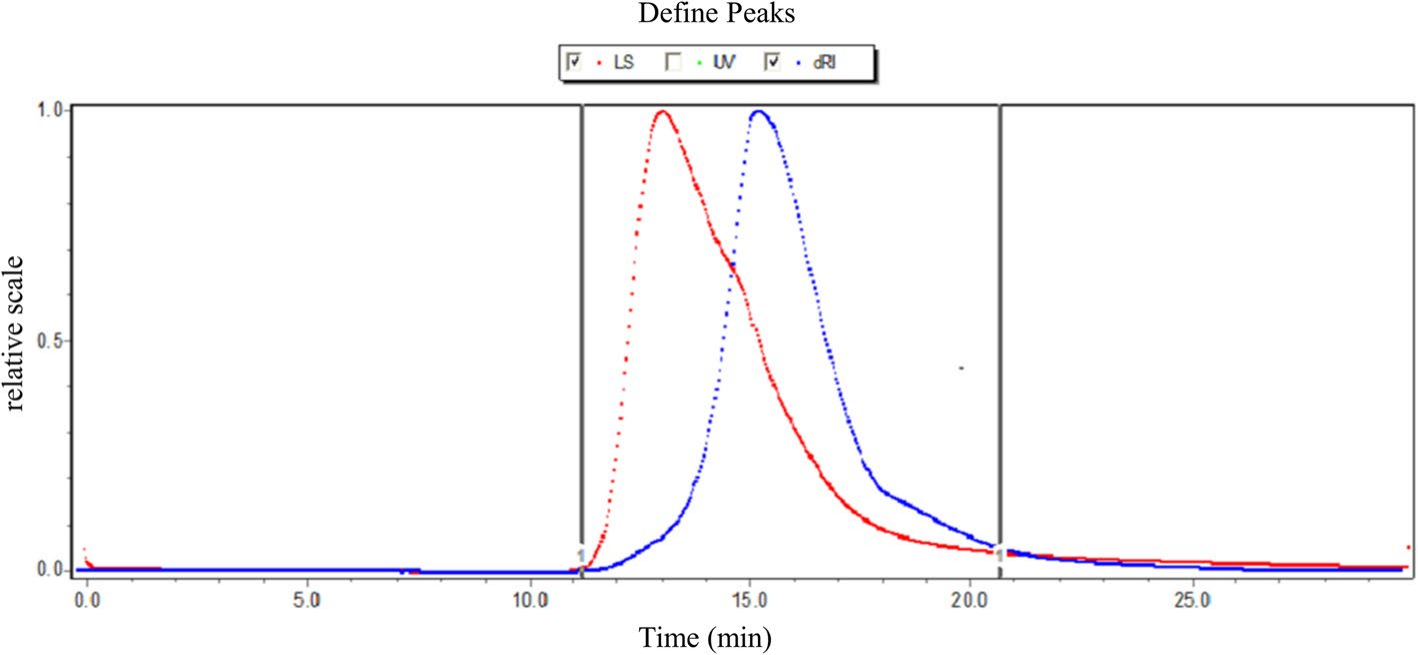
The molecular weight distribution of WFP

**Fig. 2:**
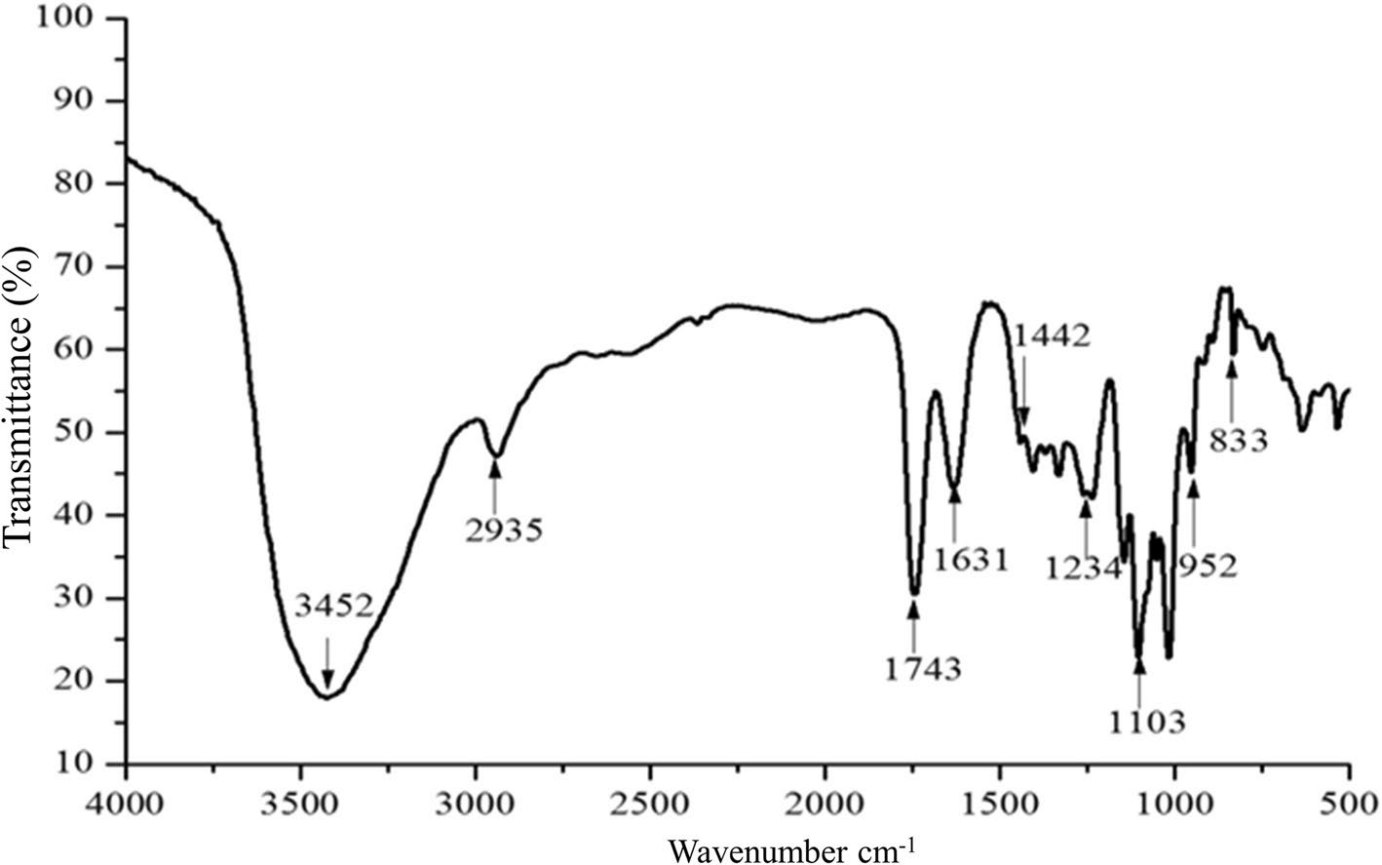
FT-IR spectra of WFP

The crude pectin (WFP) from wampee fruit is extracted by hydrochloric acid, and precipitated by ethanol. In this work, we obtained about 6.54% yield of crude pectin, which suggested the existence of abundant content of pectin. Subsequently, pectin was dissolved in water, repeated freezing and thawing, and dialyzed with different molecular weight dialysis bags. Theoretically, the molecular weight of pectin obtained by purification is more than 100 kDa. In this study, the Mw was calculated as 5.271 × 10^5^ Da (Table 1). The pectin exhibited no obvious impurity peak (Fig. 1), revealing the homogeneity. Thus, the separation method was suitable according to the result of the molecular weight and distribution of purified pectin. As shown in Table 1, the content of galacturonic acid was significantly higher than 65% of the national standard. The results showed that the quality of pectin was great. The FT-IR spectra of WFP, exhibiting various and typical absorption peaks, were performed in the region 4000 ~ 400 cm^− 1^ as shown in Fig. 2.

**Table 1:** The molecular weight and DE of wampee fruit pectin

Sample	Content (%)	Content (%)	Content (%)	DE	Gel strength	Mw
WFP	Total carbohydrate 99.5	Uronic acid 77.33	Protein Not detected	60.55	128.03	5.271 × 10^5^ Da

### Structural characterization of WFP

**Fig. 3:**
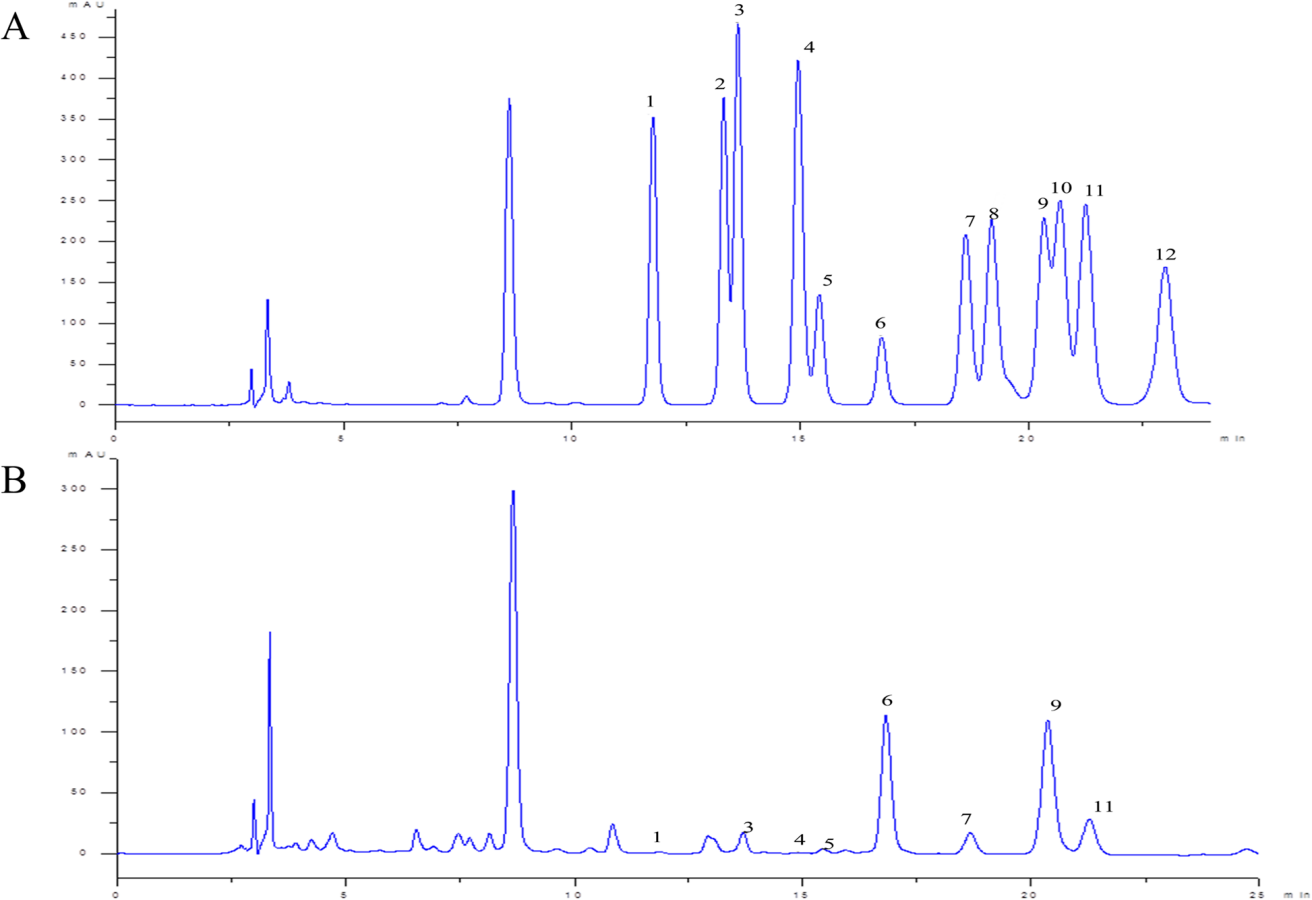
HPLC analysis of monosaccharide standards (**A**) and WFP (**B**). 1. Mannose (Man); 2. Ribose (Rib); 3. Rhamnose (Rha); 4. Glucosamine (Glu); 5. Glucuronic acid (Glu A); 6. Galacturonic acid (Gal A); 7. Glucose (Glu); 8. Galactosamine (Gal); 9. Galactose (Gal); 10. Xylose (Xyl); 11. Arabinose (Ara); 12. L-fucose (L-fuc)

**Fig. 4:**
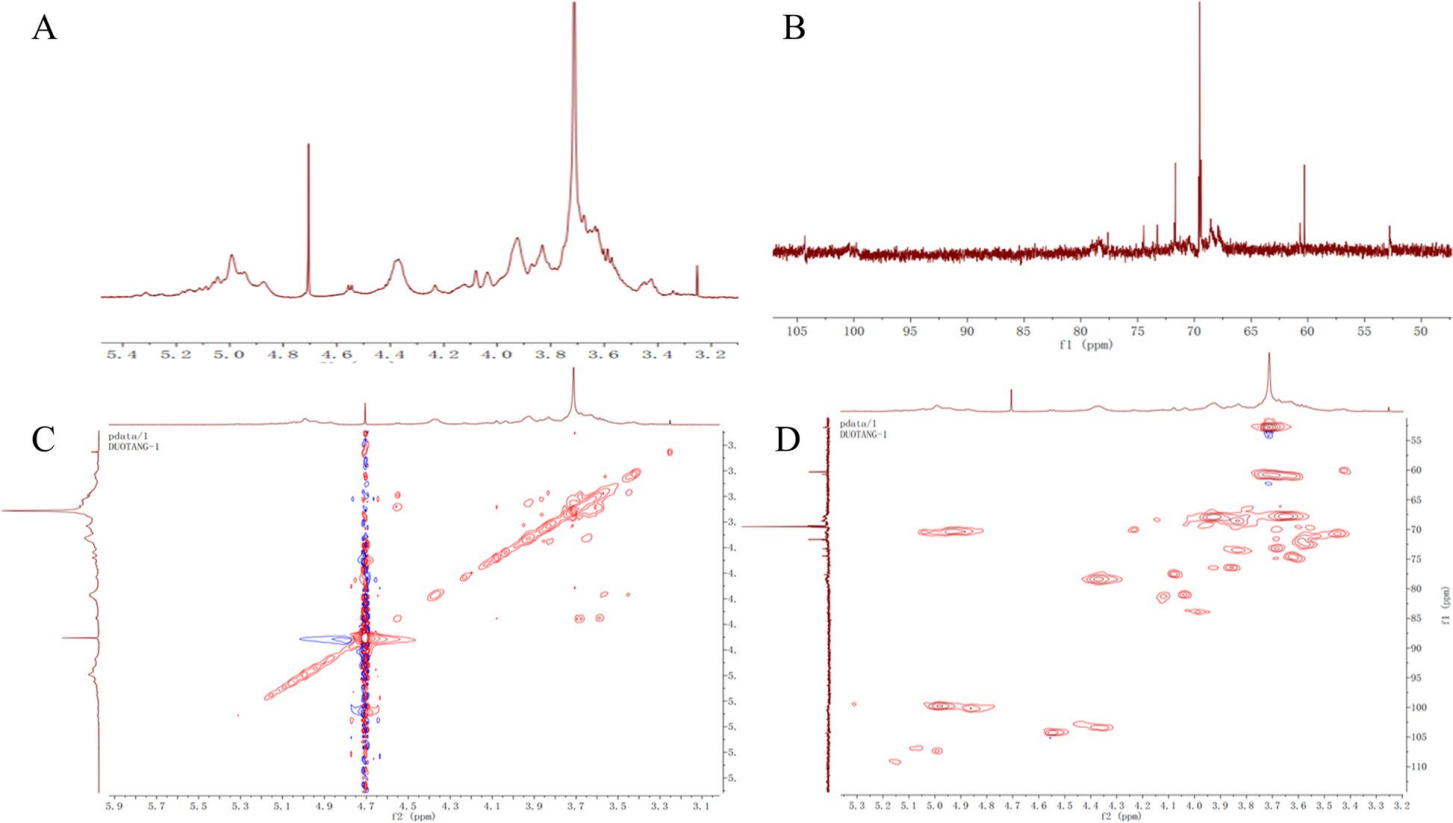
NMR of WFP: (**A**) ^1^H NMR of WFP, (**B**) ^13^C NMR of WFP, (**C**) ^1^H-^1^HCOSY of WFP, (**D**) ^1^H/^13^C-HSQC of WFP

As shown in Fig. 3 and Table 2, the content of galacturonic acid and galactose in WFP was the highest, which indicated that WFP contained an abundant content of acid sugar, and WFP was free of galactosamine, xylose, L-fucose, and glucosamine. It’s useful to gain abundant information about chemical structures and the chain configurations by the NMR spectra. The NMR spectrum of WFP was shown in Fig. 4 containing the ^1^H NMR, the ^13^C NMR of WFP, the ^1^H-^1^HCOSY, and the ^1^H/^13^C-HSQC spectrum. According to the results, the structure of sugar residues was assigned to →4)-β-D-Galp-(1→. The chemical shifts of WFP were shown in Table 3.

**Table 2:** The monosaccharide composition of WFP

Components (%w/w)	Pectin
Mannose	0.33%
Ribose	0.27%
Rhamnose	2.83%
Glucosamine	0.00
Glucuronic acid	1.10%
Galacturonic acid	43.16%
Glucose	6.75%
Galactosamine	0.00
Galactose	36.91%
Xylose	0.00
Arabinose	8.66%
L-fucose	0.00

**Table 3:** Chemical shifts of WFP

Residues	Nucleus	Chemical shifts, δ (ppm)					
		1	2	3	4	5	6
→4)-β-D-Galp-(1→	^13^C	104.32	69.63	77.61	71.67	71.94	–
	^1^H	4.56	3.56	4.37	3.45	3.59	–

### Effects of WFP on TRY activity

**Fig. 5:**
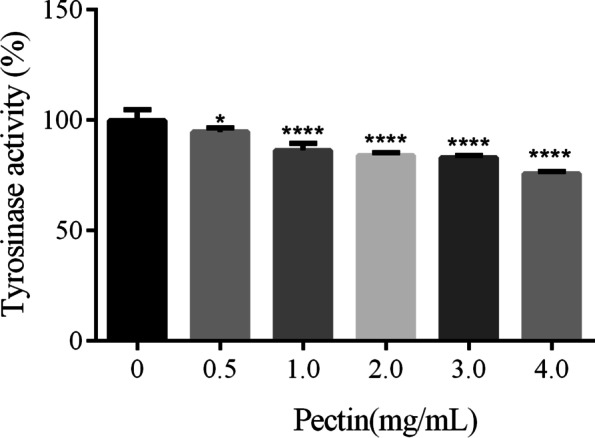
Effect of WFP on TRY activity. The results are presented as the means ± SDs from three independent experiments. ^*^P < 0.05, ^****^P < 0.0001 vs 0 group, *n* = 5

The WFP was evaluated to validate their role as tyrosinase enzyme inhibitors. L-dopa (5 mM) was used as the reaction substrate and the results obtained as inhibition (%) value are summarized in Fig. 5. Interestingly, WFP potently and dose-dependently inhibited L-dopa oxidase activities of TRY and the activity of TRY decreased after adding different concentrations of WFP to the reaction system. WFPexhibited inhibition potential against TRY at the dose of 0.5 mg/mL ~ 4 mg/mL. As shown in Fig. 5, the inhibition rate of TRY was increased from (5.27 ± 1.72) % to (24.23 ± 1.06) % with the concentrations of pectin increasing. Furthermore, TRY activity increased most obviously at the range of 0.5 mg/mL ~ 1.0 mg/mL.

### Effects of WFP on cell viability

**Fig. 6:**
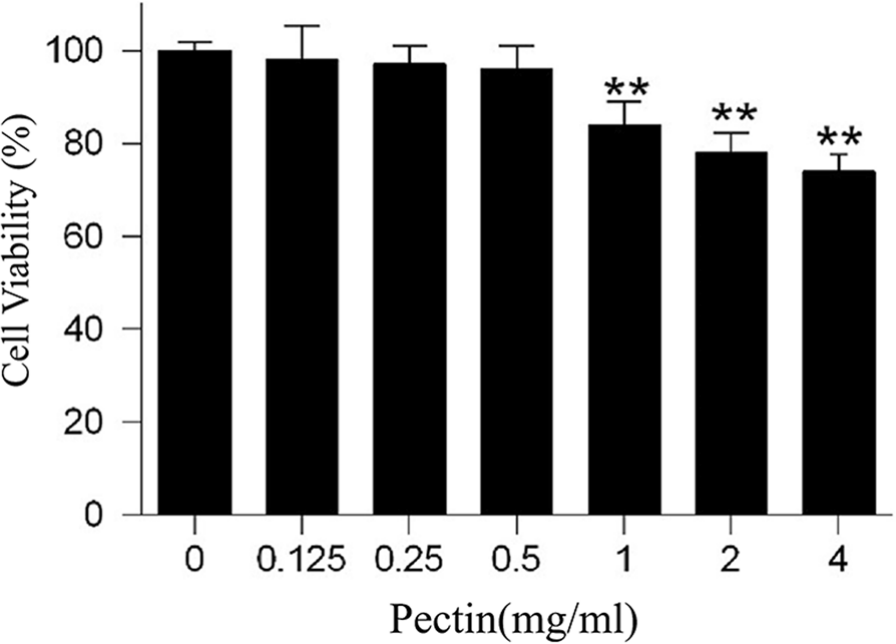
Effect of WFP on the viability of human melanoma A375 cells. The results are presented as the means ± SDs from three independent experiments. ^*^P < 0.05, ^**^P < 0.01 vs control group, *n* = 3

With the development of melanogenesis inhibitors, safety and effectiveness are the most important consideration for many applications. For the sake of determining the cytotoxic effects of WFP, α-MSH-stimulated A375 cells were treated with 0 ~ 4.0 mg/mL of WFP for 12 h. Based on the results exhibited in Fig. 6, WFP did not show cytotoxic effects in a concentration range of 0 ~ 0.5 mg/mL when the cells were treated for 12 h, while were proved to effectively suppressed cell viabilityin α-MSH-stimulated A375 cells at a range of 0.5 ~ 4.0 mg/mL in a dose-dependent manner.

### Effects of WFP on TRY activity in α-MSH stimulated A375 cells

**Fig. 7:**
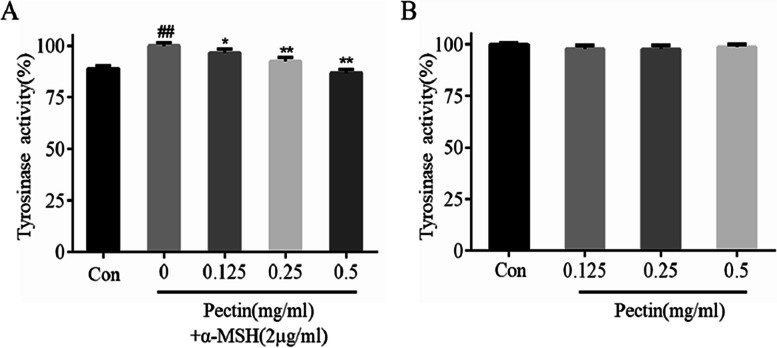
The effect of WFP on TRY activity in A375 cells induced by or without α – MSH. The results are presented as the means ± SDs from three independent experiments. **A** ^*#*^*P < 0.05,* ^*##*^*P < 0.01* vs control group; ^*^P < 0.05, ^**^P < 0.01 vs α-MSH group. **B** ^*^P < 0.05, ^**^P < 0.01 vs control group, *n* = 3

Due to TRY for melanin synthesis being a key enzyme, we investigated the effect of WFP on TRY activity in α-MSH stimulated A375 cells. The cells were treated with 2 μg/mL of α-MSH and non-cytotoxic concentrations of WFP for 12 h. As shown in Fig. 7A, TRY activity in a-MSH-stimulated A375 cells increased significantly and the difference was statistically significant (*P < 0.01*). With the concentration increasing from 0.125 mg / mL to 0.5 mg / mL, TRY activity decreased from (96.48 ± 2.03) % to (86.74 ± 1.78) %. Similarly, the cells were treated with non-cytotoxic concentrations of WFP for 12 h. As shown in Fig. 7B, the WFP slightly reduced intracellular TRY activity as compared with α-MSH-stimulated cells (*P > 0.05*). These results suggested that WFP could inhibit the production of melanin to some extent by inhibiting the activity of TRY in A375 cells.

### Effects of WFP on melanin content in α-MSH stimulated A375 cells

**Fig. 8:**
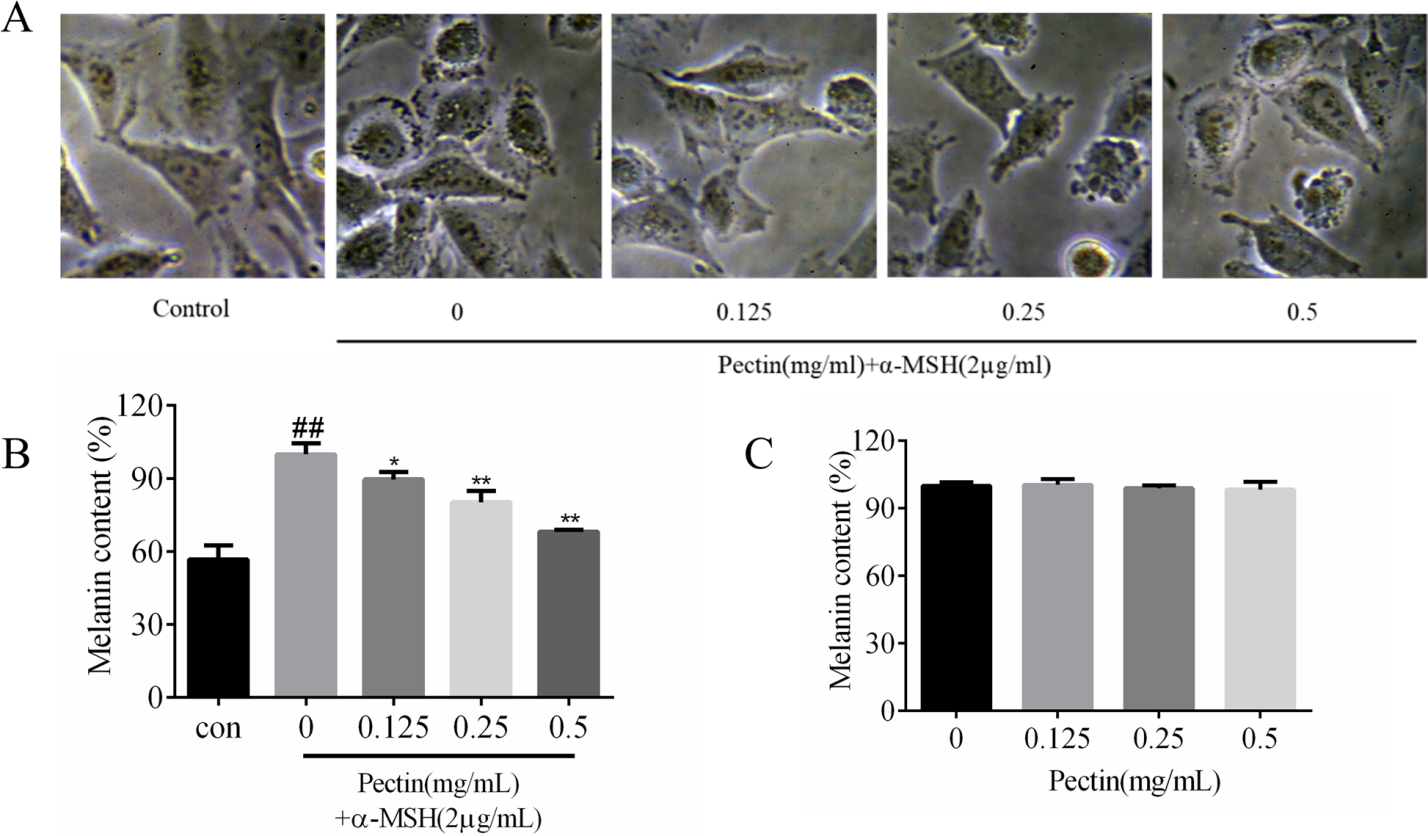
**A** The inhibition of melanogenesis induced by α - MSH in A375 cells. **B** Effects of WFP on melanin content in α-MSH stimulated A375 cells. **C** Effects of WFP on melanin content in A375 cells without α-MSH stimulation. The results are presented as the means ± SDs from three independent experiments. The results are presented as the means ± SDs from three independent experiments. ^*#*^*P < 0.05,* ^*##*^*P < 0.01* vs control group. ^*^P < 0.05, ^**^P < 0.01 vs control group, *n* = 3

To determine the influence of WFP on melanin content in the presence of α-MSH, the cells were treated with 2 μg/mL of α-MSH and non-cytotoxic concentrations of WFP for 12 h. Figure 8A showed that the amount of melanin content in α-MSH-treated cells increased significantly compared with the non-α-MSH-treated cells. After adding pectin, the melanin content of α-MSH-treated cells decreased in a dose-dependent manner, and the cell morphology did not change during the test. As shown in Fig. 8B, treatment with the WFP at the range of 0.125 ~ 0.5 mg/mL reduced the intracellular melanin levels from (89.78 ± 3.09) % to (68.28 ± 0.69) %, and the difference was statistically significant. Figure 8C showed that the anti-melanogenic activity of WFP in cells without α-MSH stimulation was not obvious. As shown in Fig. 4, the number of melanin spots increased significantly (compared with no α-MSH -treated cells), while the number of melanin spots decreased after 12 h of treatment with different concentrations of pectin, and the morphology of cells in high concentration group (0.5 mg/mL) also changed. These results suggest that WFP can suppress cellular melanin synthesis in α-MSH-induced A375 cells.

### Effects of WFP on the expression of melanogenic proteins

**Fig. 9:**
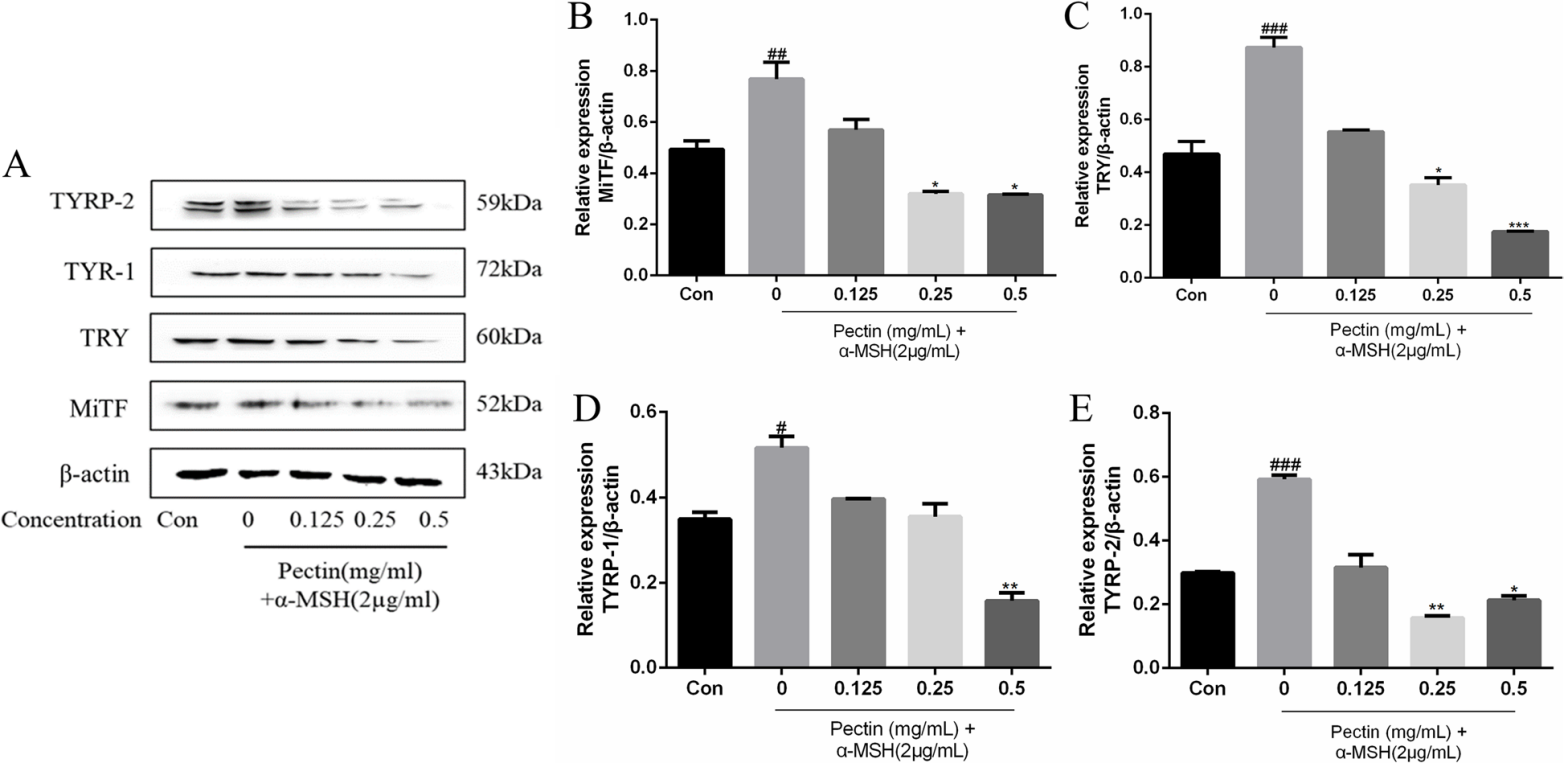
The effect of WFP on protein expression of MITF, TRY, TYRP-1 and TYRP-2 in human melanoma A375 cells induced by α – MSH. **A** The levels of MITF, TRY, TYRP-1, and TYRP-2 were determined by western blot analysis. **B-E** The results are presented as the means ± SDs from three independent experiments. ^*#*^*P < 0.05,* ^*##*^*P < 0.01* vs control group. ^*^P < 0.05, ^**^P < 0.01 vs control group

**Fig. 10:**
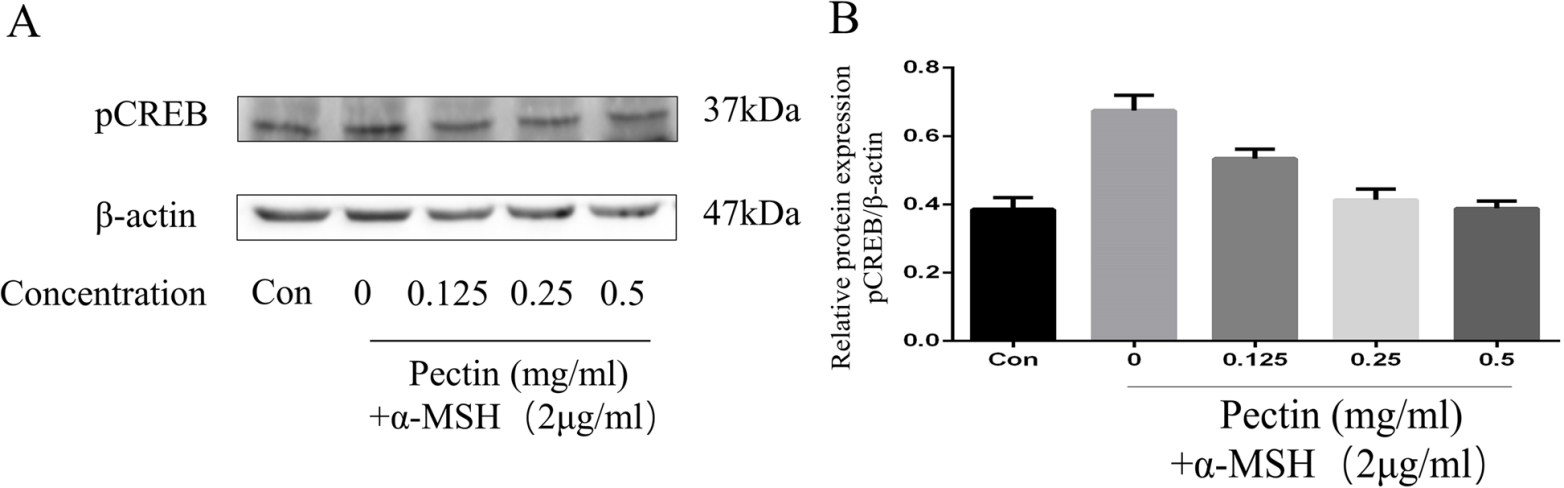
The effect of WFP on protein expression of pCREB in human melanoma A375 cells induced by α – MSH. **A** The levels of MITF, TRY, TYRP-1, and TYRP-2 were determined by western blot analysis. **B** The results are presented as the means ± SDs from three independent experiments

The effects of WFP on the expression of the melanogenic proteins MITF, TRY, TYRP-1, TYRP-2 and pCREB, were determined. A375 cells were treated with 2 μg/mL of α-MSH, and non-cytotoxic concentrations of WFP for 12 h. Protein levels of MITF, TRY, TYRP-1 and TYRP-2 were clearly increased after 12 h of treatment with α-MSH. Furthermore, MITF, TRY, TYRP-1, TYRP-2, and pCREB proteins in a-MSH stimulated A375 cells were also suppressed by 0.125 mg/mL to 0.5 mg/mL of WFP (Fig. 9 and 10). These results demonstrate that the expression of MITF, TRY, TYRP-1 and TYRP-2 in A375 cells induced by α-MSH was significantly down regulated by pectin.

## Discussion

Here we firstly reported a novel pectic polysaccharide structure, and its anti-melanogenesis effect. In the current study, the obtained characteristics of WFP identified by FT-IR spectrometric were basically consistent with the infrared characteristics of pectin reported in related literatures. In the FT-IR spectrum, the intense broad absorption peak at 3452 cm^− 1^ was considered as stretching vibration of hydroxyl groups existing in intra or inter molecular hydrogen bonds [24], and the weak absorption peaks at 2935 cm^− 1^ were attributed to the stretching vibration of C-H bond [25]. The absorption peak at 1743 cm^− 1^ was due to the vibration of the C-O bond from the methoxy group [26]. The stretching peaks at around 1631 cm^− 1^ and 1442 cm^− 1^ were ascribed to the presence of carboxyl groups in WFP. Strong absorptions at 1103 cm^− 1^ and 1018 cm^− 1^ were attributable to the glycosidic bond vibration [27]. The absorption peaks between 1234 cm^− 1^ ~ 952 cm^− 1^ could be ascribed to the presence of the pyran ring in WFP [28].

Through further experiments on GCP and the degree of esterification, we found that the composition of pectin was mainly galacturonic acid, the degree of esterification was 60.55%, and the molecular weight was 5.271 × 10^5^ Da. WFP was mainly composed of Gal A, suggesting HG was the major pectin type in these fractions [29]. Moreover, Gal A, the major component of WFP, can suppress melanin synthesis [30]. Besides, previous research has shown that the good antioxidant activity of the pectic polysaccharides produced from teamed ginseng may be related to the retention of Gal A [31]. Gal A probably played the important role of the active component of WFP on anti-melanogenesis. The NMR spectrum was employed to further confirm the obtained structure data and provide more detailed WFP structural information. In the ^1^H NMR spectrum, the regions δ 4.56 showed obvious coupling splitting and were attributed to heterotopic hydrogen signals, and ^3^J_1,2_ was 6.23. Some studies reported that the region of heterotopic hydrogen signals in the fields of 4.4 ~ 4.8 ppm was characteristic of β-configuration, which meant the glycoside bonds of WFP belonged to β-configuration [32]. The region δ 2.09 showed resonance signals, which indicated that the branch chain structure of WFP might contain the acetylated sugar residue in the ^1^H-NMR spectrum [33]. The ^13^C-NMR spectrum displayed the signals at 90 ~ 110 ppm, which were assigned to heterocephalic carbon, and the region 52.79 ppm was contributed to the methoxy group. In addition, the signals at 3.25 ppm in the ^1^H-^1^HCOSY and 52.79 ppm in the ^1^H/^13^C-HSQC were further confirmed to the presence of the methoxy group [34]. Four obvious chemical shifts were found at 3.56 ppm, 4.37 ppm, 3.45 ppm, and 3.59 ppm in the ^1^H-^1^HCOSY. These signals were attributed to H-2 to H-5. The signal at 3.83 ppm might be the resonance signal of the carboxy combination of methyl and Gal A [35, 36]. Based on the hydrogen signal fragment, the chemical shifts from C-1 to C-5 could be found in ^1^H/^13^C-HSQC. The characteristic absorption signals at 68.55 ppm and 67.85 ppm were assigned to C-2 and C-3 from Gal A residues and near 69.53 ppm was assigned to C-5 from Ara [37–39]. According to the related research, combined with the results of ^1^H and ^13^C chemical shifts and monosaccharide composition, it was speculated that the structure of sugar residues was assigned to →4)-β-D-Galp-(1→. In addition, the above results showed that WFP had a strong gelling capacity, which indicated that it was a less neutral sugar side chain pectin.

Melanin synthesis and pigmentation were considerably associated with the overactivation of the TRY enzyme [40]. Thus, the suppression of the TRY enzyme plays an important role in developing potential whitening agents. Herein, WFP could significantly inhibit mushroom TRY activity in a dose-dependent manner. For intracellular experiments, the human melanocyte line that depends on the activation of TRY pathway is a key element in efficiently evaluating the anti-melanin synthesis effect of WFP. The A375 cell line, a metastatic cell line of the human amelanotic melanoma, has been widely used as a reliable model for screening anti-melanin agents [41–43], which is dependent on TRY pathway activation [44, 45]. Firstly, thecytotoxic effect of WFPin A375 cells should be evaluated, and eliminate the influence caused by the decease of melanin synthesis due to cell numbers decline. The cell proliferation result suggested that WFP below 0.5 mg/mL were used to further evaluate anti-TRY activity in α-MSH stimulated A375 cells. Furthermore, melanin synthesis and pigmentation were associated with an unfavorable increase in α-MSH [46]. In this study, WFP significantly inhibited TRY activity of α-MSH-stimulated cells, and the change could not be exhibited in the cells non-stimulated by α-MSH. Additionally, WFP also caused a dose-dependent melanin decrease in α-MSH-stimulated cells. Thus, our result indicated that WFP decreased melanogenesis dependent on α-MSH mediated TRY activation, and the activation results from the inhibition of TRY.

**Fig. 11:**
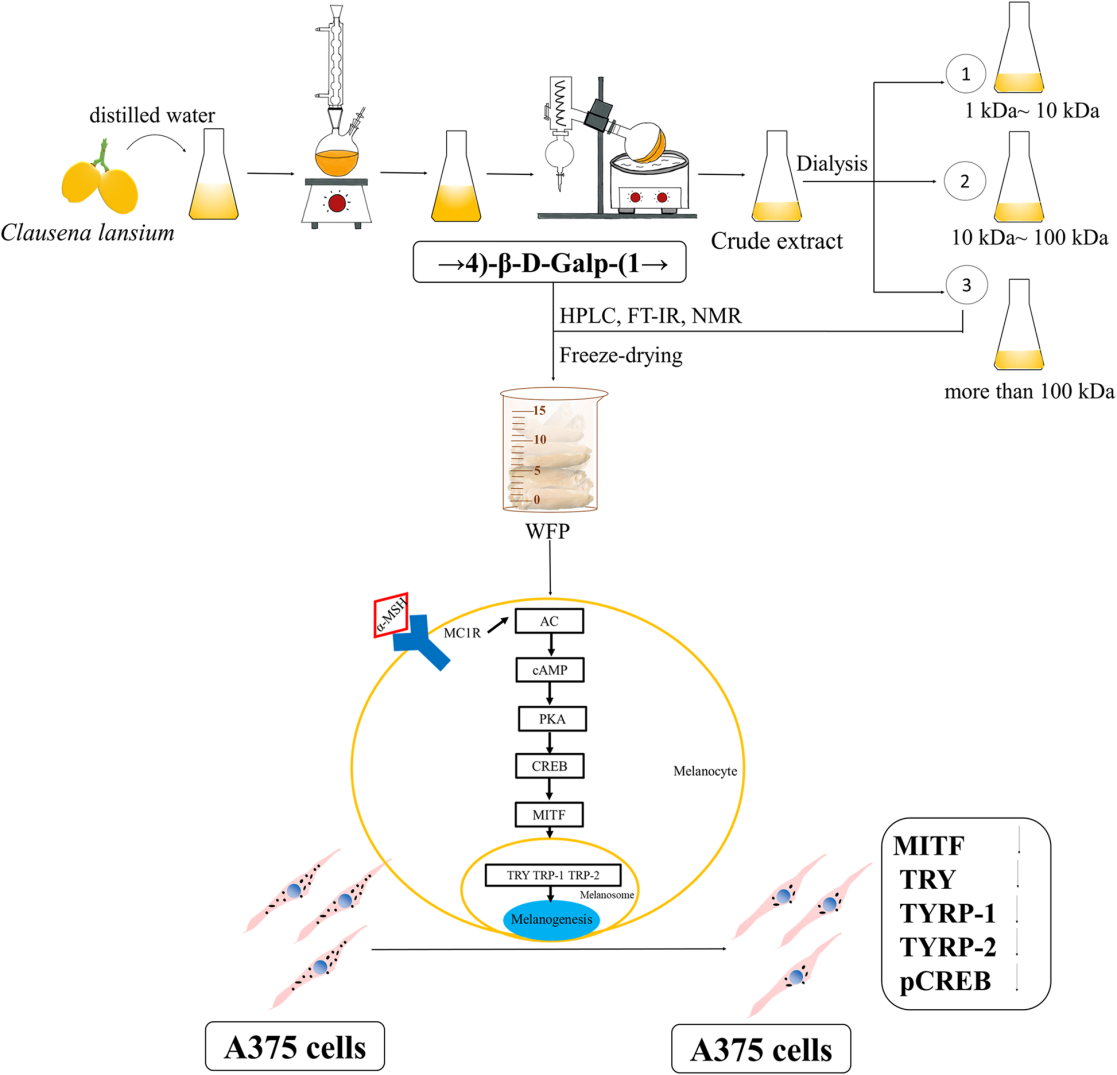
Anti-melanogenesis Effect from Wampee Fruit Pectin via α-MSH/TRY Pathway in A375 cells

It was well known that melanin synthesis was controlled by catalyzation of TRY, TYRP-1, and TYRP-2 that could be activated by α-MSH. Many studies have studied the inhibition of melanin synthesis by regulating TYRP-1 and TYRP-2 [12, 47], and the inhibition of TYRP-1 and TYRP-2 could further downregulate the stability of TRY [39]. In the study, WFP significantly downregulated the expression of TRY, TYRP-1, and TYRP-2, which were upregulation in α-MSH stimulated cells. Furthermore, microphthalmia-associated transcription factor (MITF) and cAMP-response element-binding protein (CREB) were a regulatory role in A375 cells of melanin synthesis [43]. The cAMP synthesis was dependent on α-MSH binding to MC1R, and then induced phosphorylation of CREB [17, 46]. The phosphorylation CREB activated MITF promoter to synthesize melanin [48]. Our results were consistent with those of previous studies reported that WFP significantly downregulated the expression of MITF and phosphorylation CREB in α-MSH-stimulated cells in a dose-dependent manner [47, 49].. We suggested that WFP inhibited melanin synthesis through the α-MSH mediated TRY/MITF pathway (Fig. 11), and this inhibition might be a top-down correlation reaction in the pathway.

## Conclusions

Pectin, isolated and purified from the fruits of wampee, was removed by repeated freezing and thawing, and separated and purified by dialysis. Considering the results presented in this study (monosaccharide composition, NMR, FT-IR, and linkage analyses), it is possible to suggest that the molecular weight distribution of WFP was concentrated in the range of 5.271 × 10^5^ Da, mainly composed of galacturonic acid and galactose. The results of IR and NMR showed that there was a characteristic absorption peak of sugar in pectin, and its structure may contain β-GALP glycoside bond. Furthermore, WFP showed significant inhibition of melanin synthesis and TRY activity in the α-MSH induced A375 cells model. It suggested that the anti-melanogenesis mechanism of WFP was dependent on the α-MSH/TRY pathway. The end of our study also provided a potential natural agent for the pharmaceutical and cosmeceutical industries.

## Data Availability

No additional information is supplied as a supplementary file. Additional questions or information may be obtained by contact the Corresponding author, Kefeng Wu.
